# A paradigm shift fully self-powered long-distance wireless sensing solution enabled by discharge-induced displacement current

**DOI:** 10.1126/sciadv.abi6751

**Published:** 2021-09-22

**Authors:** Haoyu Wang, Jiaqi Wang, Kuanming Yao, Jingjing Fu, Xin Xia, Ruirui Zhang, Jiyu Li, Guoqiang Xu, Lingyun Wang, Jingchao Yang, Jie Lai, Yuan Dai, Zhengyou Zhang, Anyin Li, Yuyan Zhu, Xinge Yu, Zhong Lin Wang, Yunlong Zi

**Affiliations:** 1 Department of Mechanical and Automation Engineering, The Chinese University of Hong Kong, Shatin, NT, Hong Kong, China.; 2 School of Marine Sciences, Sun Yat-Sen University, Zhuhai, Guangdong 519082, China.; 3 Department of Biomedical Engineering, City University of Hong Kong, Kowloon Tong, Kowloon, Hong Kong, China.; 4 Tencent Robotics X, Shenzhen, Guangdong 518054, China.; 5 Department of Chemistry, University of New Hampshire, Durham, NH 03824, USA.; 6 Department of Applied Biology and Chemical Technology, The Hong Kong Polytechnic University, Hung Hom, Kowloon, Hong Kong, China.; 7 Beijing Institute of Nanoenergy and Nanosystems, Chinese Academy of Sciences, Beijing 100083, China.

## Abstract

The rapid development of the Internet of Things depends on wireless devices and their network. Traditional wireless sensing and transmission technology still requires multiple modules for sensing, signal modulation, transmission, and power, making the whole system bulky, rigid, and costly. Here, we proposed a paradigm shift wireless sensing solution based on the breakdown discharge–induced displacement current. Through that, we can combine the abovementioned functional modules in a single unit of self-powered wireless sensing e-sticker (SWISE), which features a small size (down to 9 mm by 9 mm) and long effective transmission distance (>30 m) when compared to existing wireless sensing technologies. Furthermore, SWISEs have functions of multipoint motion sensing and gas detection in fully self-powered manner. This work proposes a solution for flexible self-powered wireless sensing platforms, which shows great potential for implantable and wearable electronics, robotics, health care, infrastructure monitoring, human-machine interface, virtual reality, etc.

## Teaser

An all-in-one fully self-powered wireless sensing e-sticker was designed for smart sensing, robotics, smart city, and Industry 4.0.

## INTRODUCTION

**Fig. 1.**
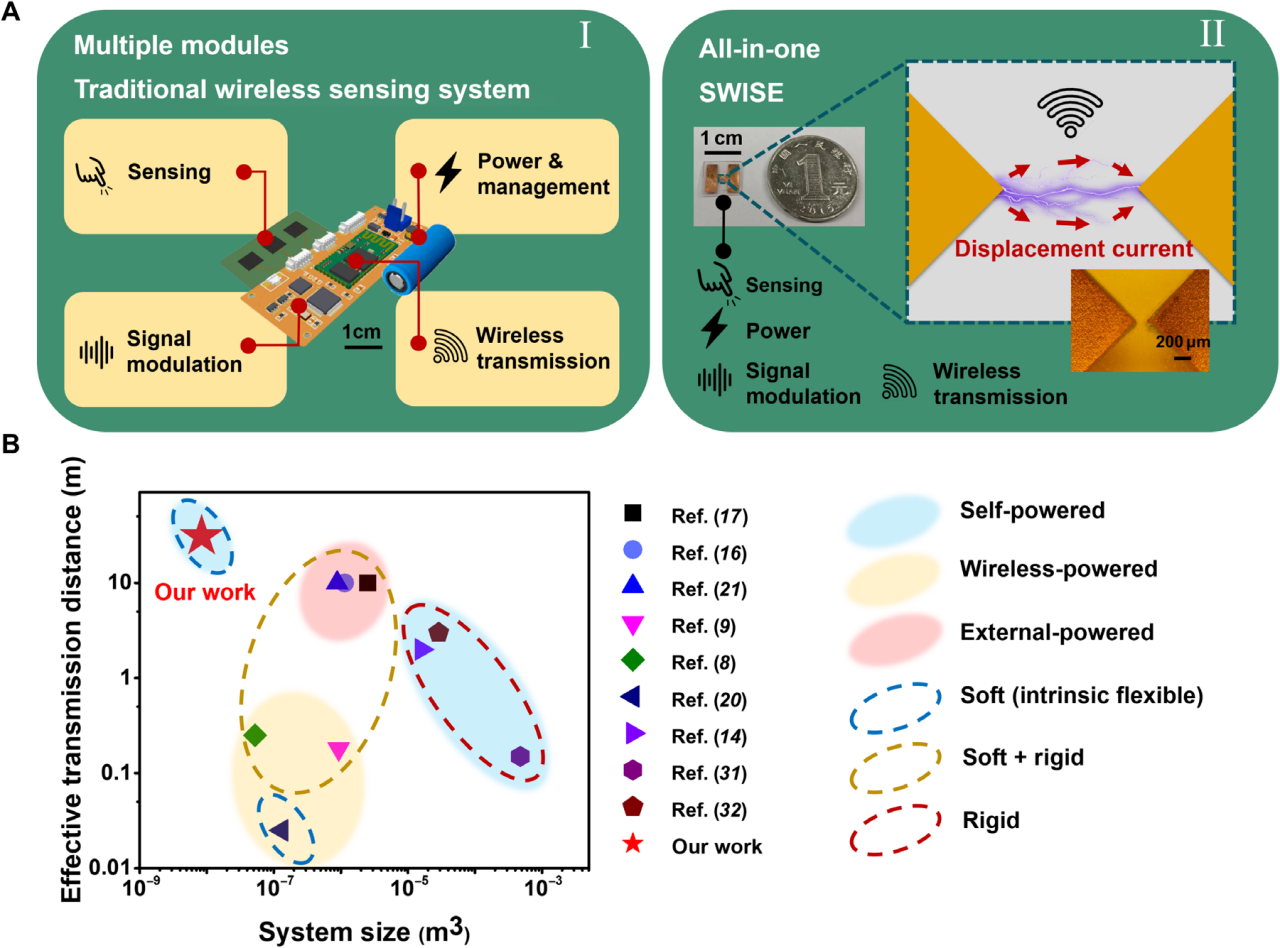
The overall illustration of the SWISE. (**A**) The comparison of the traditional wireless sensing system (I) with SWISE (II), where the SWISE has the characteristic of small, thin, light, and all-in-one. (**B**) The comparison of the SWISE with relevant research, demonstrating the smallest system size and longest effective transmission distance.

Toward the realization of the fourth Industrial Revolution, Internet of Things (IoT) that landed in the first decade of 21st century have been increasing its stupendous ability in many areas, such as to guarantee hospital supply chains in a smart way during this coronavirus disease 2019 pandemic (*1*, *2*). For unleashing the great potential of IoT in different scopes, wireless sensing and transmission based on electromagnetic (EM) waves is highly demanded as a core technology (*3*, *4*). For instance, current robotics for intelligent applications are still suffering from wiring cables with huge complexity and restricted mobility, bringing great inconvenience and obstacles in design, motion control, and maintenance (*5*–*7*). Although the wireless transmission technology has been implemented and used for over one century, its further development suffers from following challenges. First, as shown in Fig. 1A (I), the current wireless system includes modules for signal generation, power source and management, signal modulation, and transmission, with rigid and bulky electronic components (*8*–*14*). Although stretchable and flexible electronics have been developed to address the soft-rigid interface issues, most of them are still composed of intrinsically rigid devices, inducing inconveniences and limiting the application scenarios such as e-skins and implantable medical devices. Second, the total energy consumption of these electronic components is usually large, approaching milliwatt level and even watt level (*15*, *16*). Therefore, batteries or cables are used to supply the required power, which brings inconveniences in implementation and maintenance and raises issues of sustainability and environment (*17*–*21*).

To breakthrough this bottleneck, it is required to explore alternative strategies for wireless sensing, which may achieve the all-in-one unit by comprehensively containing functions of the power source and management, sensing, modulation, and transmission. A potential method may be through the emerging triboelectric nanogenerator (TENG) technology (*22*), which brings the additional displacement current term ∂***P***_**S**_/∂*t* to trigger the wireless signal generation and transmission, where ***P***_**S**_ is the polarization term brought by the TENG (*23*–*26*). Since both the mechanical energy and motion signals can be effectively captured by TENG simultaneously (*27*–*29*), no additional modules for power and sensing are needed. The power consumption for EM wave emission is usually less than 1 mW, which can be easily achieved by typical body motion energies as harvested by the TENG (*30*), making the device fully self-powered. By reducing intermediate steps through such the all-in-one configuration, it is possible to avoid additional electronic components and power consumptions to achieve the effective wireless transmission through the minimized device. However, how to create the rapidly varied ***P***_**S**_ in TENG to generate a large enough ∂***P***_**S**_/∂*t* for effective wireless signal transmission is still challenging (*31*, *32*).

Here, we proposed a paradigm shift strategy through the breakdown discharge triggered by the TENG, demonstrating a self-powered wireless sensing e-sticker (SWISE), which involves functions of all abovementioned modules in a single mini-sized unit (down to subfinger nail size, shown in Fig. 1A, II). Compared with various previous works, the SWISE generates rapidly varied polarization term ***P***_**S**_ through the breakdown discharge, which features the smallest size in volume and the longest effective transmission distance, as shown in Fig. 1B. By avoiding the additional power consumptions from intermediate steps, SWISE is fully self-powered by the captured signal as energy source without any external power input. The capabilities of multipoint motion sensing and gas sensing were achieved by distinguishing the signals generated from different design parameters and gas compositions. Because of its multipoint sensing capability, mass production of SWISEs can be demonstrated for applications as self-powered wireless keyboard and smart wristband. With the fully self-power ability, the smallest size and the longest effective transmission distance in existing wireless sensing technologies, and taking advantages of the intrinsic flexibility, low cost, and high scalability, this approach will demonstrate great potentials in applications of health care, smart home, smart city, robotics, Industry 4.0, etc.

## RESULTS

### Theory of the discharge-induced wireless signal generation

The state-of-the-art wireless signal generation can be fundamentally explained by Faraday’s law (Eq. 1) and Maxwell-Ampere’s law (Eq. 2)

$$\nabla \times E=-\frac{\partial B}{\partial t}$$ (1)

$$\nabla \times H=J+\frac{\partial D}{\partial t}$$ (2)

Here, ***E*** and ***D*** are the electric field and electric displacement field, ***B*** and ***H*** are the magnetic flux density and the magnetic field strength, respectively, and ***J*** is the current density. The state-of-the-art wireless signals are mostly initiated by the varied conduction current density ***J***, followed by transformation between electric field (***E*** or ***D***) and magnetic field (***B*** or ***H***).

Here, we put forward the paradigm shift mechanism of EM wave wireless signal generation through the additional displacement current term ∂***P***_**S**_/∂*t* as proposed by Wang *et al*. (*23*–*26*) from the triboelectric effect. Thus, Eq. 2 becomes

$$\nabla \times H=J+\varepsilon \frac{\partial E}{\partial t}+\frac{\partial P_{S}}{\partial t}$$ (3)

Therefore, to generate rapidly varied electric and magnetic fields for wireless transmission, the key is to obtain rapidly varied ***P***_**S**_ at high frequency, which can be achieved through breakdown discharge effect in our proposed SWISE. The breakdown discharge is induced by collisions between electric field–driven high-speed moving electrons and air molecules, which generate ions and new electrons, forming electron avalanche and plasma. The whole process of the breakdown discharge is illustrated in fig. S1. This plasma can be considered as collective oscillation of clustered charged particles, which can thus generate the rapidly varied displacement current and hence the high-frequency signal, ready for sensing and wireless transmission, as detailed in note S1.

### Design and mechanism of the SWISE

To achieve the discharge-induced signal generation, the SWISE is rationally designed as Fig. 1A (II) and fig. S2A, where two mirrored symmetric metal electrodes with the sharp tips for discharge were sandwiched between a substrate film and a tribo-charge layer film. The intrinsically flexible materials: fluorinated ethylene propylene (FEP) films and polydimethylsiloxane (PDMS) were used as the tribo-charge film and the substrate, respectively, with the detailed fabrication methods shown in Materials and Methods and fig. S2B. The total thickness of the device was down to 95 μm. The gap distance between the two electrodes was controlled to be 10 to 500 μm, as identified in the microscope. The small features of the electrodes can be fabricated by the nonlithography methods through the laser-cut mask, which greatly reduces the cost. In this work, both the nonlithography and photolithography methods were used for fabricating different SWISE devices. Then, the SWISE has the characteristics of thin (down to 95 μm) (fig. S2C, I), small (down to 9 mm by 9 mm), light (down to 16 mg) (fig. S2C, II), soft, deformable (fig. S2C, III), and flexible.

**Fig. 2.**
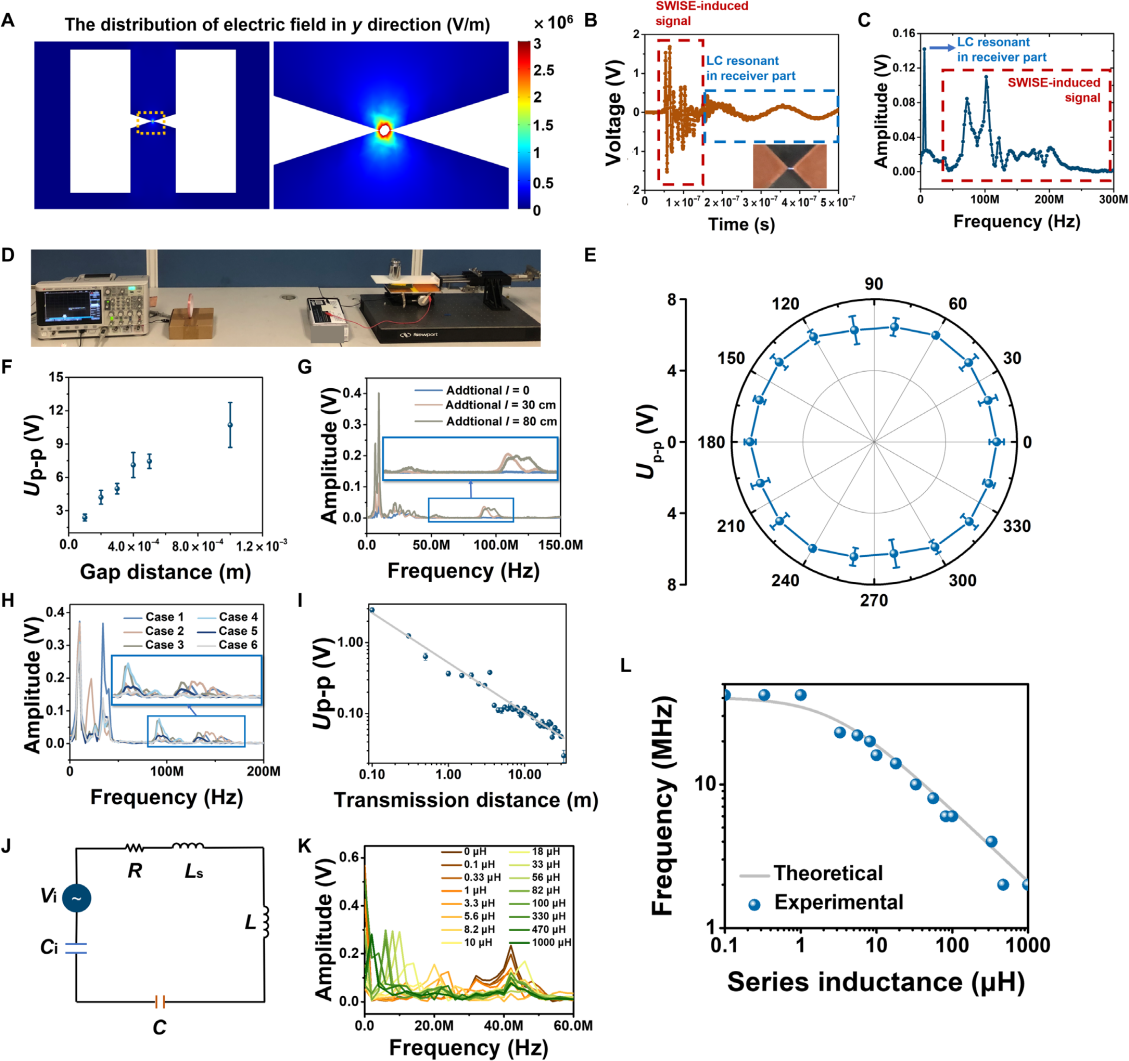
The systematic study of the SWISE-induced signal and electrical model of SWISE. (**A**) The COMSOL simulation of the SWISE’s electric field distribution in breakdown situation. (**B** and **C**) The SWISE-induced signal in time and frequency domains. (**D**) An overall illustration of the testing system. Photo credit: Haoyu Wang, Department of Mechanical and Automation Engineering, The Chinese University of Hong Kong. (**E**) The relationship between the received signal’s peak-to-peak voltage and the receiver coil’s rotation degree. (**F**) The relationship between the peak-to-peak voltage of the received signal and the gap distance between electrodes of breakdown discharger. (**G**) The received signal with different wire lengths connecting the FS-TENG and the breakdown discharger in frequency domain. (**H**) The received signal with different space conductor distribution cases in frequency domain. (**I**) The relationship between the peak-to-peak voltage of the received signal and the distance between the breakdown discharger and receiver. (**J**) The electrical model of the SWISE. (**K**) The relationship between the output signal and different external inductance in frequency domain. (**L**) The relationship between the base frequency of the output signal and external inductance.

Driven by gentle finger sliding, the SWISE can directly convert the input motion signal to EM signal through a discharge process without any external power sources, demonstrating the fully self-power ability, shown in fig. S2D. When the SWISE was triggered, the triboelectrification generated the negative charge in the tribo-charge layer. Because of the electrostatically induced charges, the electric field was generated between electrodes, with the highest value around the sharp tips (*33*), as shown by the COMSOL simulation result in Fig. 2A. The strong electric field generated the breakdown discharge, with the amplitude and rise time determined by the environment and structural factors in SWISE. Such the breakdown discharge caused the high-frequency displacement current and hence induced the wireless EM signal. Compared with the conventional high-intensity electrostatic discharge that may bring ignition hazard and damage/interfere electronic components (*34*), the SWISE has demonstrated fine EM signal generation through the discharge confined in microscale gaps and sealed cavity with a small charge amount and energy, which is relatively safe.

### Experimental characterizations of the SWISE

The generated wireless signal can be captured and measured by a remote coil connecting to an oscilloscope as the receiver. The time response of a typical signal is illustrated in Fig. 2B, and its frequency response is shown in Fig. 2C using fast Fourier transform, where the signal spectrum was distributed in several hundred megahertz, mainly in the very high-frequency (VHF) band, while the resonant frequency in the receiver was concentrated on around 10 MHz. As indicated by the measured current passing through the SWISE, the period of this discharge-induced displacement current oscillation is about 10^−9^ to 10^−6^ s (fig. S3), which is consistent with the measured EM wave’s frequency band. The breakdown discharge spark was observed between the tips of electrodes while emitting the EM waves, as shown in the inset of Fig. 2B. With the characteristic of high sensitivity, the signal from a subfinger nail size SWISE (shown in fig. S2C, II) can be detected as well, shown in movie S1.

Systematic investigations were conducted to evaluate characteristics of the generated signal by the SWISE, as shown in Fig. 2D. To conveniently study the impact of various factors, the experiments were conducted on a freestanding sliding TENG (FS-TENG) connecting to two tip-shaped electrodes for discharge (called as the breakdown discharger), which is equivalent to a SWISE. The FS-TENG consists of two copper electrodes and an FEP layer, as shown in fig. S4. A nitrile butadiene rubber (NBR) film slides on the FEP layer for triboelectrification and electrostatically induces voltage and electric field between electrodes. The breakdown dischargers were built on a printed circuit board to ensure the accuracy of the gap distance, ranging from 100 μm to 1 cm (fig. S5). As fixed on the optical platform, a linear motor was used to trigger the FS-TENG with the well-controlled moving distance of up to 120 mm. When the FS-TENG was triggered by sliding the motion part, the electric field was generated to enable breakdown discharges. The receiver was kept identical with the resonance frequency of around 10 MHz. Here, experiments were conducted to prove the high repeatability and consistency of the received wireless signals, as shown in figs. S6 and S7. On the basis of the experiments, it is demonstrated that the SWISE can transmit the wireless signals omnidirectionally, where the signal intensity detected in each direction is nearly identical as shown in Fig. 2E.

The influences of the major factors (summarized in table S1) on the signal characteristics were concluded in Fig. 2, F to I, and fig. S8. Here, we conducted control experiments, where only one parameter varied, while all others kept unchanged in each measurement. The initial condition was set at a transmission distance *D* = 60 cm, voltage drop *U* = 2000 V, electrode gap distance *d* = 100 μm, temperature *T* = 23° to 24°C, humidity *H* = 60 to 65%, wire length *l* = 60 cm, and negative motion direction (from right to left). The amplitude of the received signal increased when *d* increased, with no impact on the frequency spectrum, as shown in Fig. 2F and fig. S8A. The *l* has affected both the signal’s amplitude and the frequency spectrum, as shown in Fig. 2G, which may be resulted from the varied capacitance and inductance in the equivalent circuit. Similar phenomenon was found by changing the space conductor distribution, as shown in Fig. 2H, with different cases detailed in note S2. By adjusting *U*, the amplitude of the signal increased when the *U* increased, while there was little influence on the frequency spectrum, as shown in fig. S8, B and C. The motion direction in FS-TENG was demonstrated to affect the initial phase and amplitude of transmitted signals, where the initial phase of the positive direction (from left to right) was $\frac{\pi }{2}$, while that of the negative direction (from right to left) is $-\frac{\pi }{2}$, as shown in fig. S8 (D and E). Besides, to figure out the relationship between peak-to-peak voltage of the received signal and transmission distance *D*, a long-distance experiment platform was set, as shown in fig. S9. The peak-to-peak voltage of the received signals decreased with the increased *D*, as shown in Fig. 2I, demonstrating the long effective transmission distance of over 30 m.

### Electrical model of the SWISE and the base frequency tuning

The electrical model of the SWISE operation can be equivalent to an inductor-resistor-capacitor (LRC) circuit including the capacitance between electrodes, as well as the resistance, inductance, and additional capacitance from the spatial conductors and circuits, as shown in Fig. 2J. The detailed model was described by the note S3. To further study such the electrical model, an additional inductance *L*_s_ was series-connected to the SWISE equivalent circuit to study the relationship between *L*_s_ and the frequency spectrum of the received EM signal, with the experimental setup detailed in Materials and Methods. By adding different *L*_s_, there was an obvious peak shift phenomenon in the base frequency *f* of the received signal, as shown in Fig. 2K, with the plot of the *f* versus *L*_s_ shown in Fig. 2L. Apart from the breakdown discharger, the total system capacitance *C*_e_, including *C*_i_ from the TENG and *C* from the circuits and spatial inductors, can be measured as 5.719 pF, and the system inductance *L* was measured as 2.713 μH. The base frequency *f* of the SWISE equivalent circuit in the low-frequency spectrum can be calculated by

$$f=\frac{1}{2\pi \sqrt{(L_{s}+L)C_{e}}}$$ (4)

With the parameters given by the measured results, the theoretical *f*-*L*_s_ plot was depicted in Fig. 2L, which is highly consistent with the experimental results. These results confirmed the electrical model of the SWISE, which also provides a convenient method to tune the spectrum of the SWISE to achieve multipoint wireless sensing and transmission.

### The gas environment experiment of SWISE

**Fig. 3.**
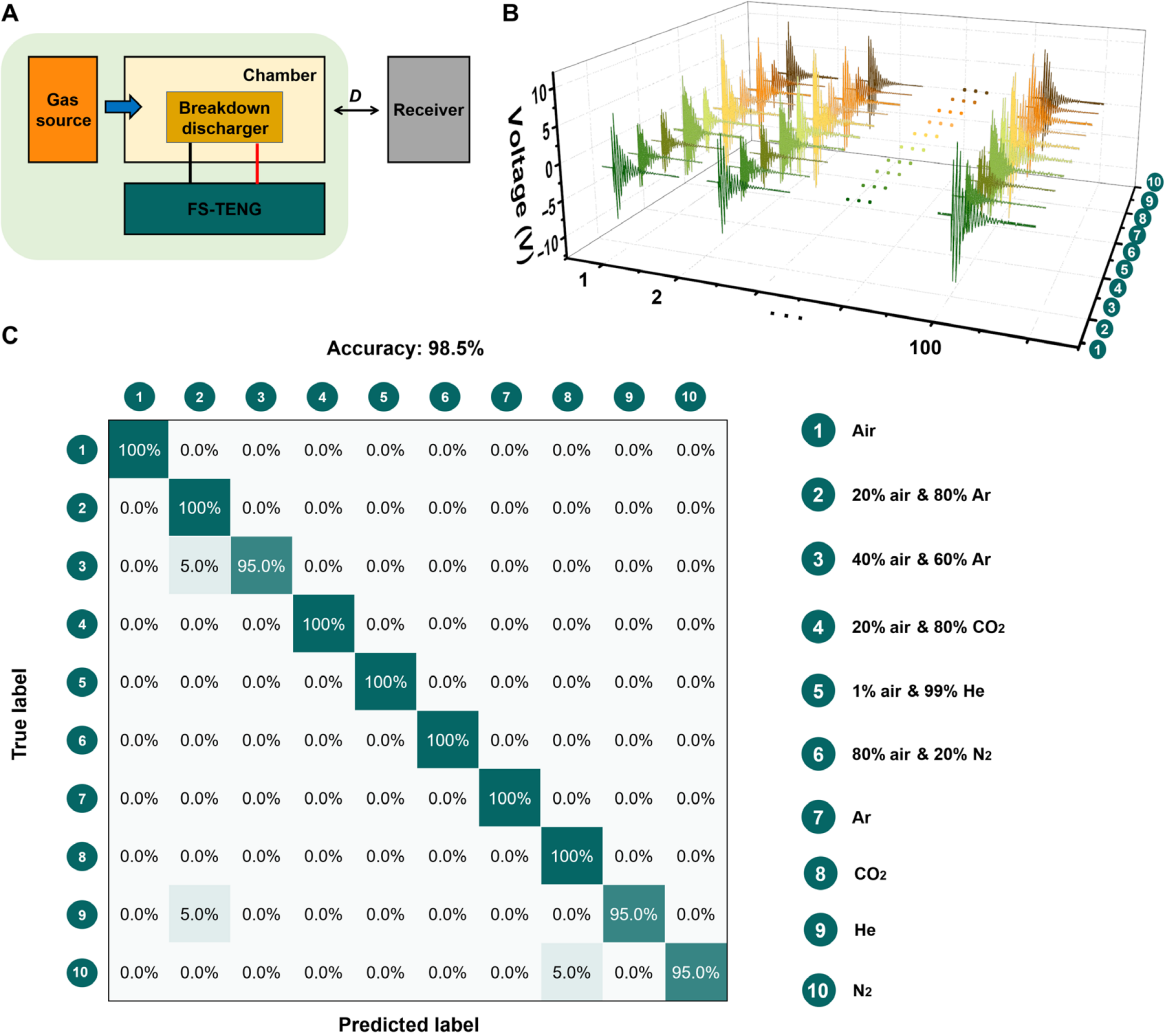
The gas environment experiment of SWISE. (**A**) The experiment platform to recognize different gas environments. (**B**) Three-dimensional plots of the breakdown discharger outputs responding to different gas environments [as listed in the table inset of (C)]. (**C**) The confusion matrix of the deep learning outcome.

In addition, environmental factors as listed in table S1 were demonstrated to make a great impact on the discharge behavior (*35*–*37*), which may also affect the wireless signals. Here, the influence of the gas type was systematically studied, with the experiment platform shown in Fig. 3A. It has been demonstrated previously that the discharge type and intensity may be distinctly different in various gas environments (*38*). To create a pure gas environment, the breakdown discharger was placed in a chamber, as driven by the FS-TENG. A process flow of the experiment was designed to ensure the purity of the target gas, as shown in fig. S10. Four types of pure gas and six types of mix-gas as labeled in Fig. 3C were tested, and the typical signal waveforms of these gases were shown in Fig. 3B and fig. S11. In this process, the data from 10 types of gas environments were collected by repeating the breakdown discharge in them, where 100 sets of data were collected from each type of gas environment. Each set of data was a voltage to time waveform, which contains around 2500 data points, as shown in Fig. 3B. As an effective method for classification and recognition, the deep learning method was used to process these complicated datasets and extract features by automatically generated algorithms (*39*–*41*). The bi-directional long short-term memory (Bi-LSTM) model (*42*) was built to analyze the data from different gas environments to realize the gas recognition. The 100 datasets from each gas environment were randomly divided into two groups, with 80 sets for training and 20 sets for testing. By training the Bi-LSTM model, the results showed highly positive predictive value and true positive rate for recognizing each gas, where the total recognition accuracy reached 98.5%, as shown in Fig. 3C. On the basis of the results, we predict that the wireless signals from multiple SWISEs with different gas compositions in the cavity can be distinguished through the deep learning method, which may enable the capability of gas sensing, as well as the multipoint motion sensing toward SWISE sensing array.

### Demonstration of SWISE for wireless motion sensing

**Fig. 4.**
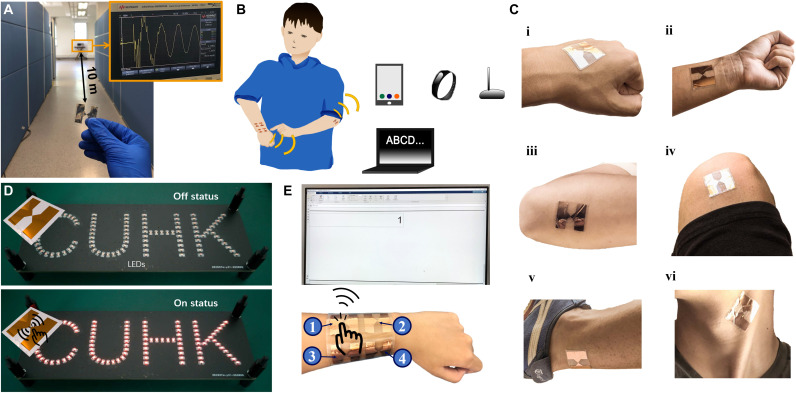
The demonstrations of SWISE. (**A**) The wireless signal can be detected with a distance of over 10 m. Photo credit: Haoyu Wang, Department of Mechanical and Automation Engineering, The Chinese University of Hong Kong. (**B**) The overall illustration of SWISE-based electronic skin and smart wristband. (**C**) Demonstrated SWISE-based electronic skin was easily integrated with skin. (i) Hand. (ii) Arm. (iii) Elbow. (iv) Leg. (v) Ankle. (vi) Neck. (**D**) Photograph of LEDs powered by the SWISE based self-powered wireless body motion electronic skin sensing system in an off and on status. Photo credit: Ruirui Zhang, Tencent Robotics X. (**E**) Photograph of the soft keyboard and smart wristband system, where the signal 1 was transformed. Photo credit: Haoyu Wang, Department of Mechanical and Automation Engineering, The Chinese University of Hong Kong.

With the characteristics of lightweight, high sensitivity, low cost, soft, and deformable, the SWISE can be widely used in signal sensing and transmission, with no additional electrical power supplies required. We demonstrated two self-powered wireless sensing applications of the SWISE as below. First, we prepared the SWISE as an electronic skin to detect motion and transmit discharge-induced EM wave signals instantaneously with the advantages of long transmission distance. We demonstrated that the wireless EM signal can be detected by the receiver with a long transmission distance of over 10 m, as shown in Fig. 4A and movie S2, where the SWISE was driven by gentle finger motions. To further prove the high sensitivity, a SWISE was attached in different locations of the human body to detect body motions (Fig. 4, B and C, and fig. S12A). A coil, a signal processing circuit, and a light-emitting diode (LED)–based lighting system were connected together to illustrate the wirelessly transmitted signal. When there was no motion triggering on SWISE, no signal transmitted, and the LEDs kept off (Fig. 4D). With finger-sliding motions, a wireless signal was generated and transmitted to turn the LEDs on (Fig. 4D and movie S3) with the high sensitivity.

### Demonstration of self-powered wireless soft keyboard and smart wristband

To further demonstrate the multipoint sensing ability of SWISEs toward human-machine interface, a self-powered wireless soft keyboard was developed by integrating multiple SWISE units (Fig. 4B and fig. S12B). As a proof of concept, four keys were fabricated, where a polyethylene terephthalate (PET) film was used as the base, with four SWISEs with different gap distance working as the four keys. By adjusting the location and structural parameters of the SWISE, the amplitude and spectrum band can be varied to distinguish the signal from each key (detailed in fig. S13). By using local area network (LAN), the collected signals can be transferred from the oscilloscope to the computer, where the MATLAB platform was used to translate the detected signal into “1,” “2,” “3,” and “4,” respectively (movie S4). Thus, the SWISE realized the function of a fully self-powered wireless keyboard. Furthermore, as one of the notable applications in wearable electronics, the SWISE-based multisignal transmission wristband was demonstrated, shown in Fig. 4E and movie S5.

### Comparison between SWISE and existing technologies

The state-of-the-art wireless sensing and transmission methods are summarized and compared with SWISE as shown in Table 1 and Fig. 1B. By comparison, the external powered methods such as Bluetooth suffer from the demands of rigid electronics and continuous power supply from batteries (*16*, *17*, *21*). For wireless-powered methods such as near-field communication (NFC) and radio frequency identification (RFID), an adjacent external wireless power and signal source is usually required, which greatly limits their effective transmission distance (<0.3 m) (*8*, *9*, *20*). Existing self-powered methods by using TENGs or other energy harvesters to power external wireless transmitters (marked as “self-powered type I”) are usually integrated from multiple modules as described above, which results in a large system size with rigid components (*14*). Wireless transmission from the TENG-generated nondischarge displacement current (marked as “self-powered type II”) is limited by the low frequency (<1 kHz) generation and the relatively short effective transmission distance (~3 m in maximum), due to the slow changing rate of ***P***_**S**_ (*31*, *32*). As a comparison, in our work, the SWISE (marked as “self-powered type III”) features the smallest size in volume and the longest effective transmission distance. Through reducing the intermediate steps and electronic components, the transit time and the consumed power are greatly reduced, and thus, the self-powered wireless transmission becomes more effective with a small size. These features are highly favorable in future applications.

**Table 1.** The comparison of the SWISE with existing wireless sensing and transmission methods.

**Reference**	**Wireless transmission** **method**	**Power supply**	**Flexibility of** **components**	**System size (m^3^)**	**Effective transmission** **distance (m)**
(*17*)	Bluetooth	External powered	Soft + rigid	2.52 × 10^−6^	10
(*16*)	Bluetooth	External powered	Soft + rigid	1.14 × 10^−6^	10
(*21*)	Bluetooth	External powered	Soft + rigid	8.82 × 10^−7^	10
(*9*)	NFC	Wireless powered	Soft + rigid	9.31 × 10^−7^	1.8 × 10^−1^
(*8*)	NFC	Wireless powered	Soft + rigid	5.25 × 10^−8^	2.5 × 10^−1^
(*20*)	RFID	Wireless powered	Soft	1.30 × 10^−7^	2.5 × 10^−2^
(*14*)	Magnetic resonance	Self-powered (type I)	Rigid	1.59 × 10^−5^	2
(*31*)	Nondischarge displacement current	Self-powered (type II)	Rigid	4.76 × 10^−4^	1.5 × 10^−1^
(*32*)	Nondischarge displacement current	Self-powered (type II)	Rigid	2.88 × 10^−5^	3
Our work	Discharge displacement current	Self-powered (type III)	Soft	7.70 × 10^−9^	30

## DISCUSSION

This work presented a paradigm shift strategy for EM wave generation and self-powered wireless sensing through the discharge-induced displacement current toward the all-in-one fully self-powered wireless sensing and transmission unit: SWISE. With the reduced intermediate steps through this all-in-one configuration, the fully self-powered SWISE features the smallest size and the longest effective transmission distance (>30 m) compared to state-of-the-art devices. The systematic investigations of the SWISE revealed the major influential factors on the transmitted signal, where the signal’s amplitude and frequency spectrum can be controlled, enabling the multipoint sensing ability. The frequency spectrum of the SWISE is also very sensitive to the gas environment, enabling the possibility of gas sensing. On the basis of that, a self-powered wireless flexible keyboard and a smart wristband were fabricated to detect and transmit signals from multiple keys. The SWISE demonstrates advantages of fully self-powered, small size, long-distance transmission, lightweight, soft, thin, and high sensitivity, which may enable potential applications in wearable and implantable devices, robotics, biomedical applications, human-machine interface, infrastructural monitoring, etc.

## MATERIALS AND METHODS

### Fabrication of the SWISE through nonlithography method

A Kapton sheet (70 μm thick), with the same shape of SWISE’s mold serving as the sacrificial layer, was attached to an FEP film (50 μm thick). An electrode layer (copper or silver with 300 nm thick) was deposited on the FEP film with the laser-cut Kapton mask at the speed of 0.2 nm/s by the electron beam evaporation process (pressure of 8 × 10^−6^ torr and temperature of 30°C). Then, the Kapton layer was removed from the FEP film. Last, another FEP film (40 μm thick) was pasted to the substrate layer and used as the tribo-charge layer.

### Fabrication of the SWISE through the lithography method

The fabrication started on a flat PDMS substrate with a cross-link ratio of 10:1. The FEP film (75 mm by 75 mm square shape, 40-μm thickness) was gently laminated onto the PDMS and removed all bubbles between them to make sure they were closely attached. Then, the surface of FEP was cleaned by acetone, deionized water, and plasma cleaner subsequently to enhance the wettability of the surface. The electrode pattern was defined by photolithography and liftoff process. First, the negative photoresist (AZ 2070) is spin-coated onto the surface (3000 rpm) and prebaked at 110°C on a hotplate for 90 s. Then, the photoresist is exposed to ultraviolet (UV) light for 5 s using a mask aligner (URE-2000/35AL deep UV, Institute of Optics and Electronics, Chinese Academy of Sciences), developed in AZ 300MIF developer for 10 s and followed with a postbake at 110°C for 3 min to form the desired patterns. Then, the Cr/Cu (20 nm/200 nm) electrode layer was deposited onto the FEP surface with photoresist by electron beam evaporation system (EBS-500F, Junsun Tech Co. Ltd.) subsequently. Extra metal film outside the desired pattern is then lifted off by immersing the FEP and temporary substrate in acetone to remove the underlying photoresist. The gap between two electrodes was first covered by a Kapton tape before spin-coating one 50-μm layer of soft PDMS (cross-link ratio, 20:1), and then the tape was removed after PDMS being cured at 80°C for 15 min in an oven, and last, a tiny blank area was formed. The surface of another layer of PDMS (30 μm) was treated in plasma for 2 min and then attached onto the blank area followed by 80°C heating for 5 min to form strong chemical bonding for encapsulation. Since the micro cavity chamber was formed in between the electrode gap, peeling off the device from the temporary PDMS supporting substrate to realize the thin, soft SWISE device, which can be perfectly integrated with skin surface.

### Fabrication and characterization of FS-TENG

Two copper tapes (95 mm by 150 mm) were pasted on an acrylic plate (200 mm by 150 mm) as the electrodes, and two wires were connected with the two electrodes separately. An FEP film was attached on the top of the copper tape layer as the negatively charged material. An NBR block (95 mm by 146 mm by 25 mm) was made by attaching an NBR film to an acrylic sheet as the positively charged material. When the relative movement happened between the FEP film and NBR film, a high voltage was generated between the two electrodes by the coupling effects of triboelectrification and electrostatic induction. Here, a linear motor was used to control the motion of the FS-TENG. The transferred charge and output voltage of the FS-TENG were measured using electrometers (Keithley 6514). Because of the limited voltage measurement range, the Keithley 6514 was connected with a 5-gigohm resistor in series to measure the current by a parallel branch to the FS-TENG. The voltage *U* can be calculated as

U=IR

where *I* and *R* are the measured current and series resistor, respectively.

### The receiver system

A Cu coil (wire diameter of 250 μm, with 10 turns to form a coil with the diameter of 10 cm) with the inductance value of ~22.5 μH [measured by inductor-capacitor (LC) meter] was used as the receiver coil. By using a probe, the Cu coil was connected with an oscilloscope (Keysight DSOX2014A) to measure the received signal. The trigger mode with the rising edge trigger and 20-mV trigger level was set to detect the SWISE’s signal.

### Electric field simulation of the SWISE

COMSOL Multiphysics was used to study the electric field distribution of the SWISE in breakdown situation. The SWISE was modeled with a size of 3 cm by 3 cm and a gap distance of 100 μm, and the material copper was chosen. Then, the SWISE was placed into an air condition. The transferred charge is defined as *q* = 40 nC, and threshold breakdown electric field in the air is *E*_ba_ = 3 × 10^6^ V/m.

### The details of the Bi-LSTM model for gas recognition

As an architecture of the recurrent neural network, the Bi-LSTM is well-suitable to process, recognize, and make prediction from time series data. There were 50 neurons in each direction of the Bi-LSTM model, and then it was connected to a fully connected layer with 32 neurons. After processing by batch normalization, processed data were activated by ReLU function and connected to a fully connected layer with 20 neurons. After activated by the softmax function, the output result can be got.

### The experiment setup of the electrical model and base frequency tuning of SWISE

Here, another experiment platform was found to avoid the influence from the receiver system (LC resonant between the oscilloscope and the receiver coil) and optical platform (considered as a big parallel capacitance to reduce the frequency spectrum of the receiver EM signal). Here, a monopole antenna was used to replace the receiver coil, and the FS-TENG was placed away from the optical platform. As the EM signal induced by SWISE mainly distributed in VHF band, the dimension of the receiver antenna can affect the amplitude of the received signal without influence to the frequency spectrum.

### The self-powered wireless body motion electronic skin sensing system

The self-powered wireless body motion electronic skin sensing system is composed of a SWISE, a receiver coil, a signal process circuit, and an LED lighting system. A Cu coil (wire diameter of 400 μm, with 15 turns to form a coil with an inner diameter of 30 mm and an outer diameter of 52 mm) worked as the receiver coil to detect the signal from the SWISE. A four-stage amplifier circuit [with the Op amp ADA4891-2, direct-current (dc) power source output voltage *U* = 6 V] was used to process and amplify the received signal, since the high frequency signal of SWISE decayed quickly. The amplified signal was used to drive the group of LEDs (MHT192CRCT) in parallel.

### SWISE-based soft keyboard and smart wristband transmission and receiving system

The SWISE-based soft keyboard and smart wristband transmission and receiving system are composed of a soft keyboard (PET film as substrate and four SWISEs with different gap distance), a smart wristband (PET film as substrate based and four SWISEs with different gap distance), a Cu receiver coil (wire diameter of 250 μm, with 10 turns to form a coil with the diameter of 10 cm), an oscilloscope (Keysight DSOX2014A), and a signal processing and display part (based on the commercial software MATLAB R2020a). Each signal from SWISE can be detected by the Cu receiver coil and the oscilloscope (trigger mode). By using the LAN, the signals can be sent from the oscilloscope to the MATLAB-Simulink in real time (block Query Instrument was used). By analyzing each SWISE signal’s amplitude, the detected signal was translated into 1, 2, 3, and 4, respectively.

## Supplementary Materials

### This PDF file includes:

Figs. S1 to S13 Table S1 Notes S1 to S3 Legends for movies S1 to S5 References
