# Supplementary material for: A paradigm shift fully self-powered long-distance wireless sensing solution enabled by discharge-induced displacement current
Source: Sci Adv. 2021 Sep 22;7(39):eabi6751. doi: 10.1126/sciadv.abi6751 (PMC8457664; doi:10.1126/sciadv.abi6751)
Supplement: Supplementary file 1 — Figs. S1 to S13 Table S1 Notes S1 to S3 Legends for movies S1 to S5 References [file sciadv.abi6751_sm.pdf]

Science Advances
AAAS

Supplementary Materials for

A paradigm shift fully self-powered long-distance wireless sensing solution enabled by discharge-induced displacement current

Haoyu Wang, Jiaqi Wang, Kuanming Yao, Jingjing Fu, Xin Xia, Ruirui Zhang, Jiyu Li, Guoqiang Xu, Lingyun Wang, Jingchao Yang, Jie Lai, Yuan Dai*, Zhengyou Zhang, Anyin Li, Yuyan Zhu, Xinge Yu, Zhong Lin Wang*, Yunlong Zi*

*Corresponding author. Email: jessiedai@tencent.com (Y.D.); zhong.wang@mse.gatech.edu (Z.L.W.); ylzi@cuhk.edu.hk (Y.Z.)

Published 22 September 2021, *Sci. Adv.* **7**, eabi6751 (2021)
DOI: 10.1126/sciadv.abi6751

The PDF file includes:

Figs. S1 to S13
Table S1
Notes S1 to S3
Legends for movies S1 to S5
References

Other Supplementary Material for this manuscript includes the following:

Movies S1 to S5

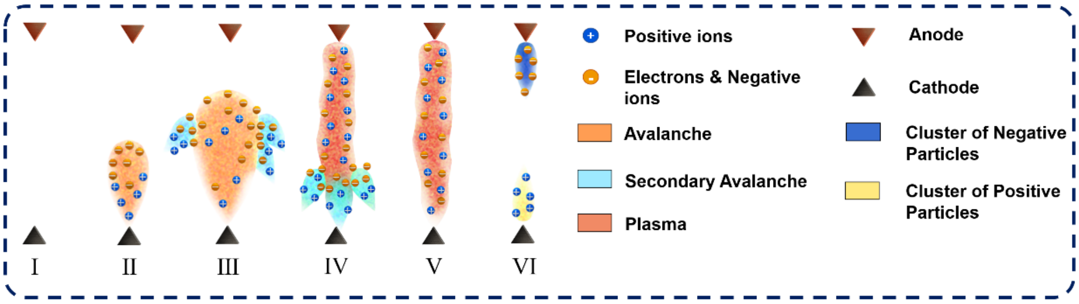

Fig. S1. The whole process of breakdown discharge.

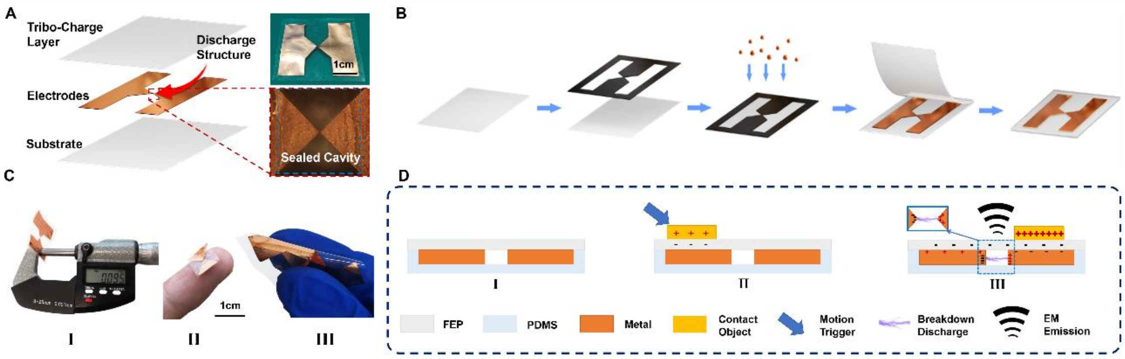

Fig. S2. The structure, fabrication process, and working process of the SWISE. (**A**) The structure and photograph of the SWISE, where the gap structure and sealed cavity was shown by microscope. (**B**) The non-lithography fabrication process of the SWISE. The electron beam evaporation process was used to fabricate the metal layer on the FEP layer. (**C**) The flexible SWISE has the characteristics of (I) thin, (II) small, and (III) deformable. (**D**) The working process of the SWISE.

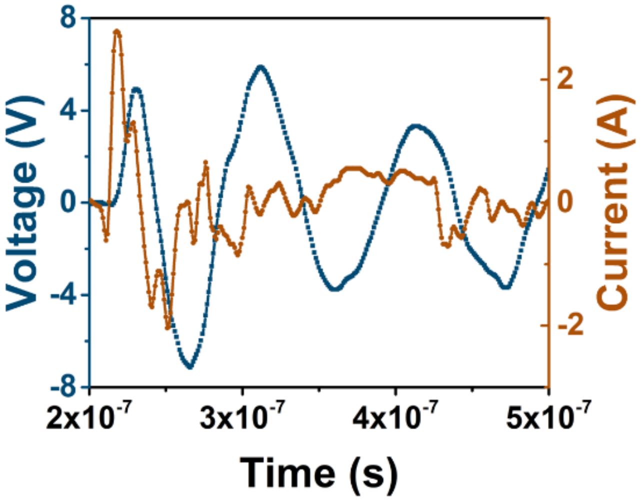

Fig. S3. The simultaneously measurement result of the transmitted signal (dark blue) and the current in the SWISE (dark orange).

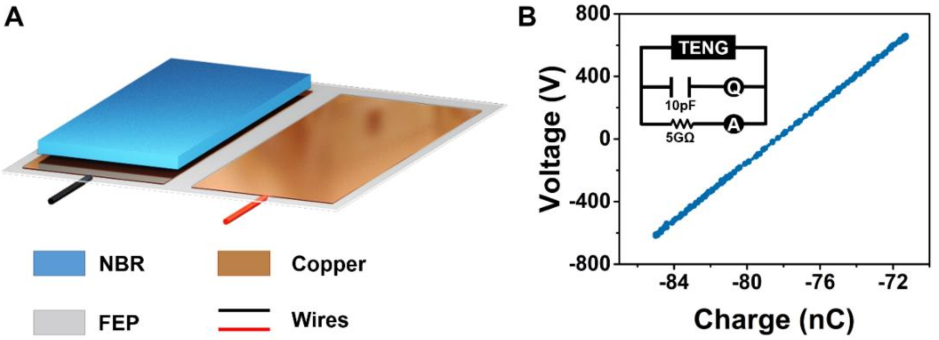

Fig. S4. The illustration of FS-TENG. (a) The structure of the FS-TENG. (b) The output characteristic of FS-TENG with a load of 10 pF capacitor in zero pressure force condition.

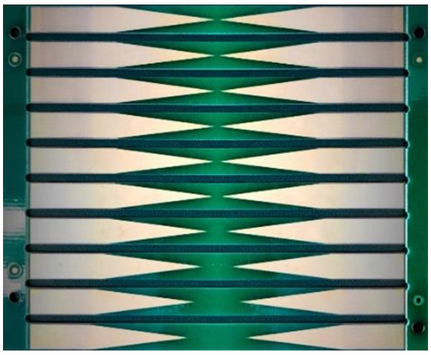

Fig. S5. The photograph of the group of breakdown dischargers. Photo Credit: Haoyu Wang, The Chinese University of Hong Kong.

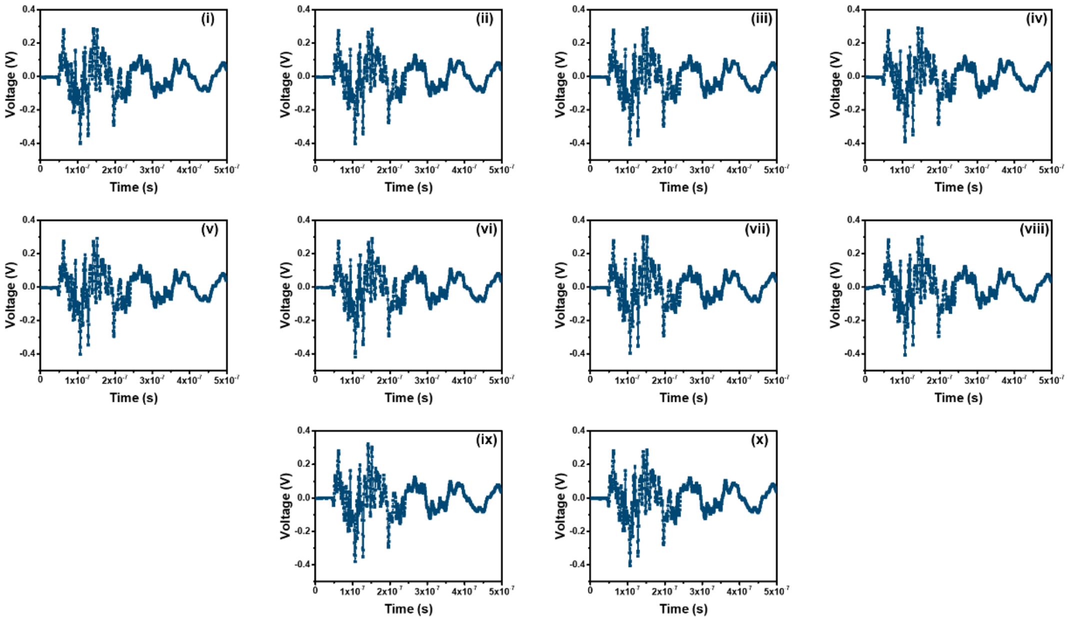

Fig. S6. The measured SWISE-induced signal of ten consecutive sliding tests in time domain.

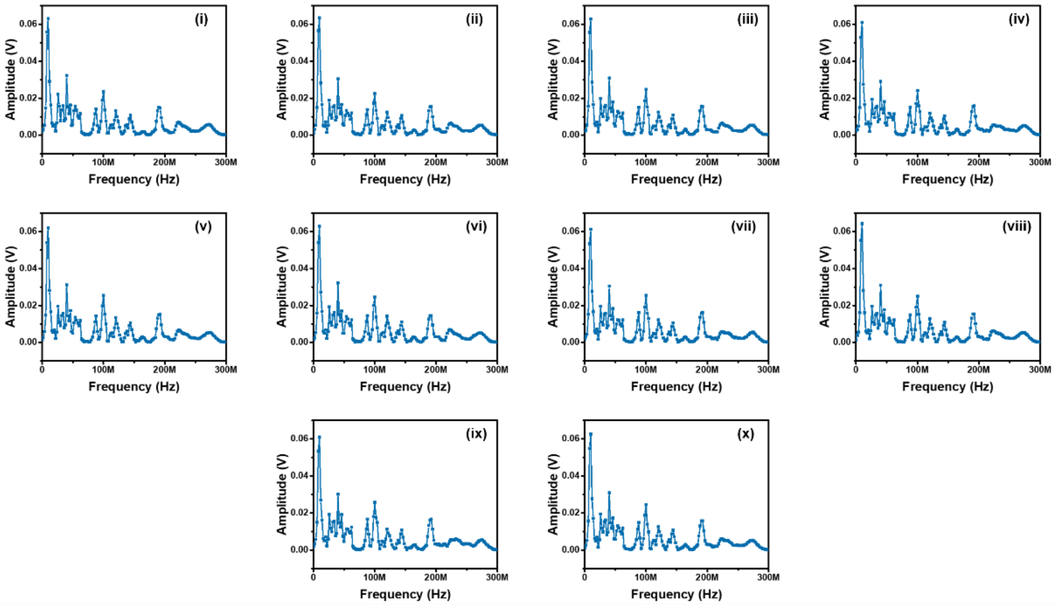

Fig. S7. The measured SWISE-induced signal of ten consecutive sliding tests in frequency domain.

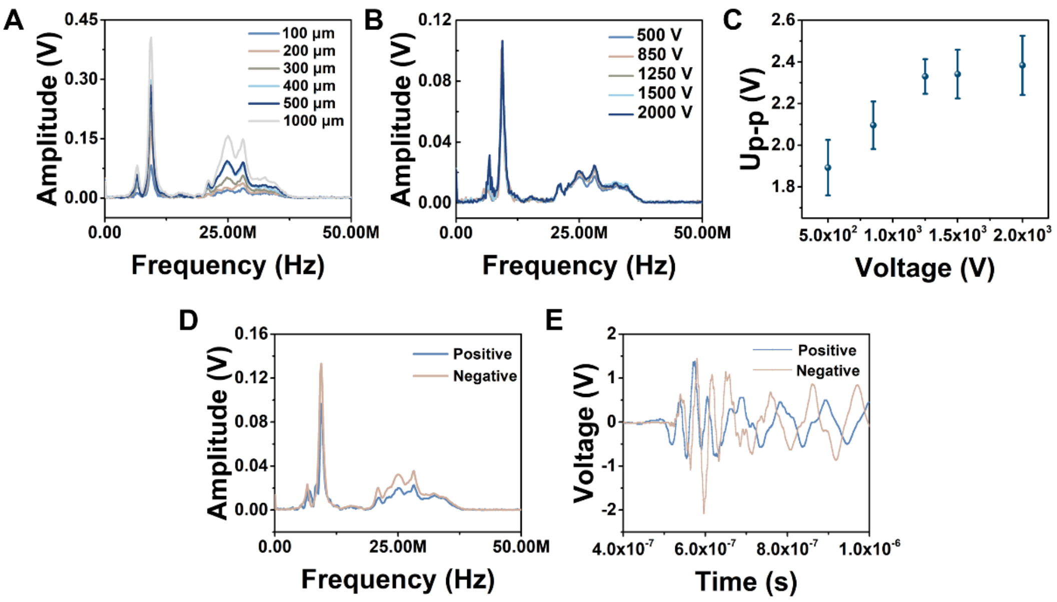

Fig. S8. The systematic study of the SWISE-induced signal. (**A**) The received signal with different gap distances between electrodes of breakdown discharger in frequency domain. (**B**) The received signal with different voltage drops on the breakdown discharger in frequency domain. (**C**) The relationship between the peak-to-peak voltage of the received signal and the voltage drops on breakdown discharger. (**D**) (**E**) The received signal with different motion directions in FS-TENG in frequency domain and time domain.

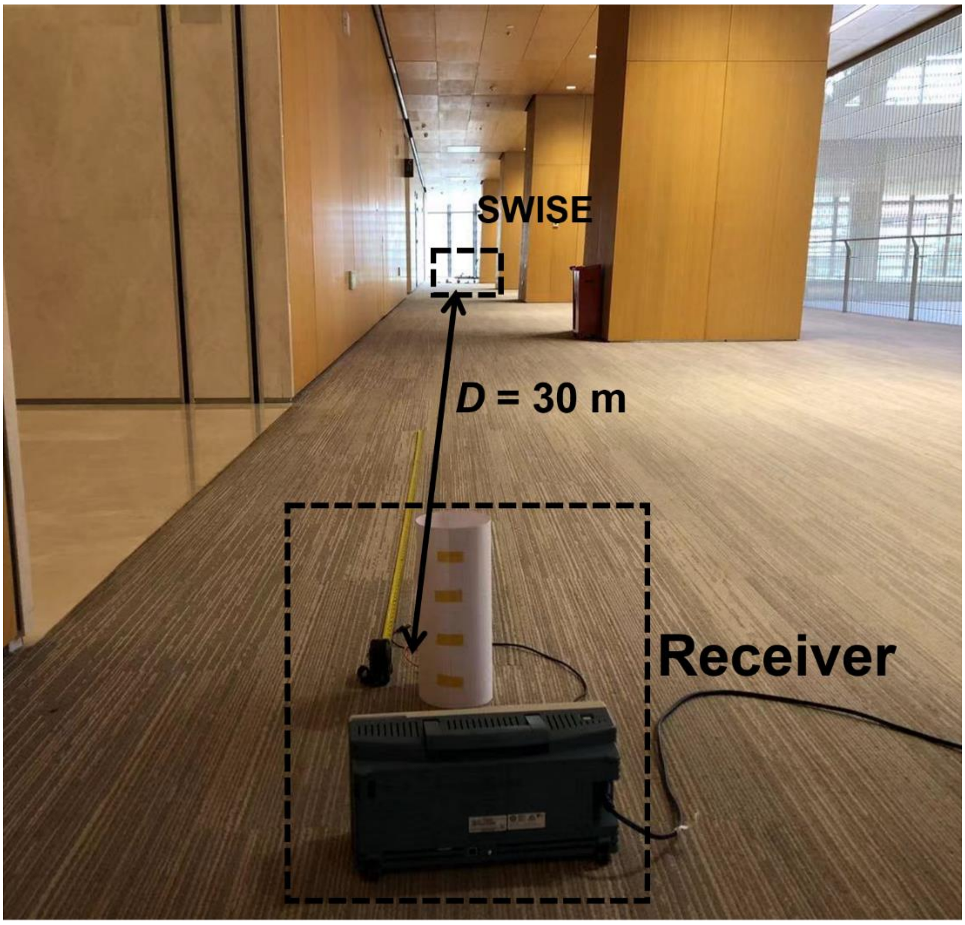

Fig. S9. The long-distance experiment platform of SWISE. Photo Credit: Haoyu Wang, The Chinese University of Hong Kong.

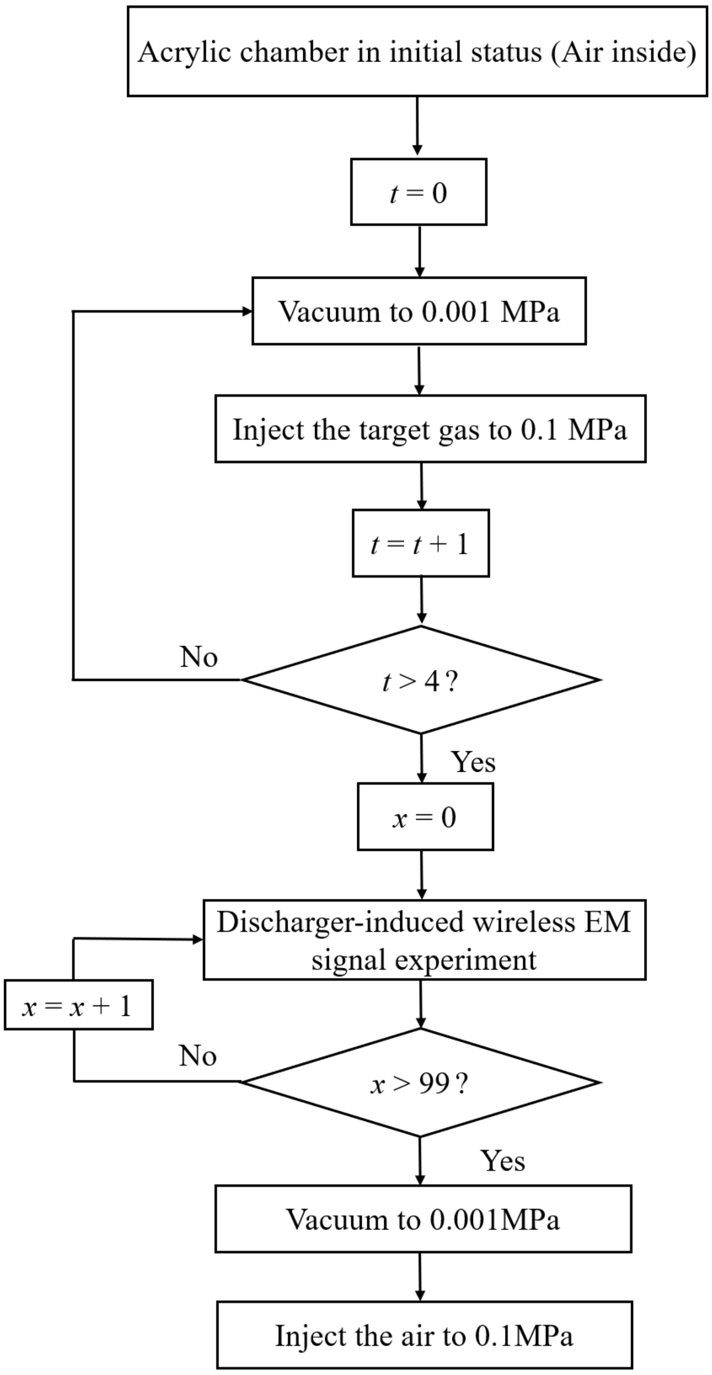

Fig. S10. The process flow of the gas environment experiment.

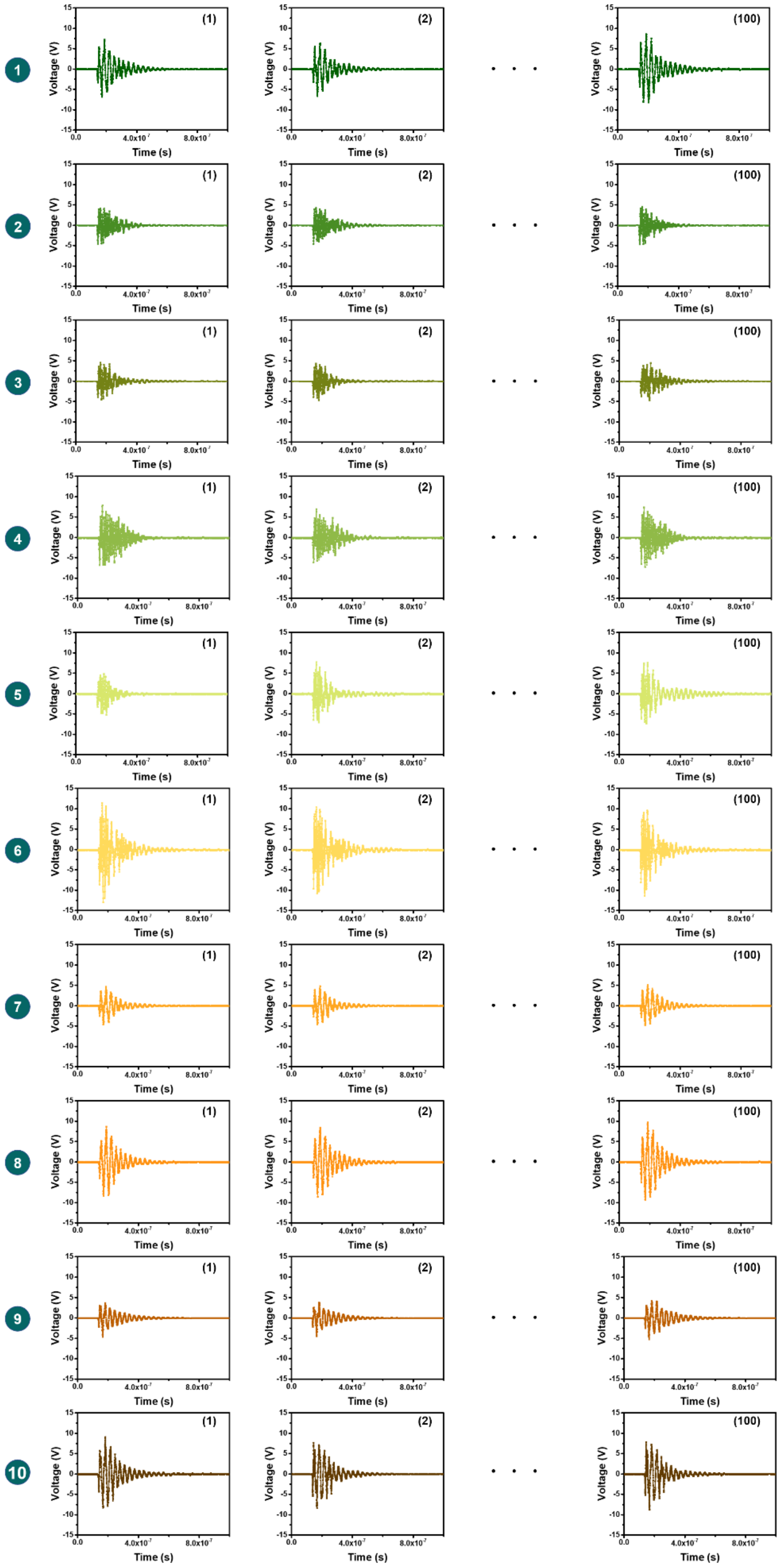

Fig. S11. 2D plots of the breakdown discharger outputs responding to different gas environments (as listed in the table inset of Fig. 3C).

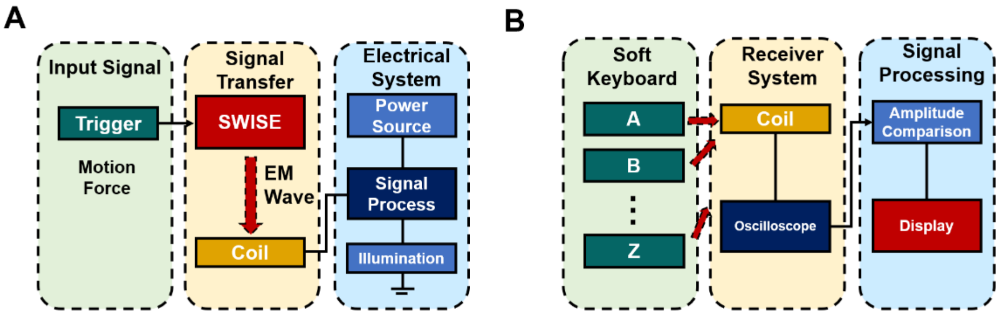

Fig. S12. Schematic diagram of the demonstration systems. (**A**) The electronic skin motion sensing system. (**B**) The self-powered wireless soft keyboard.

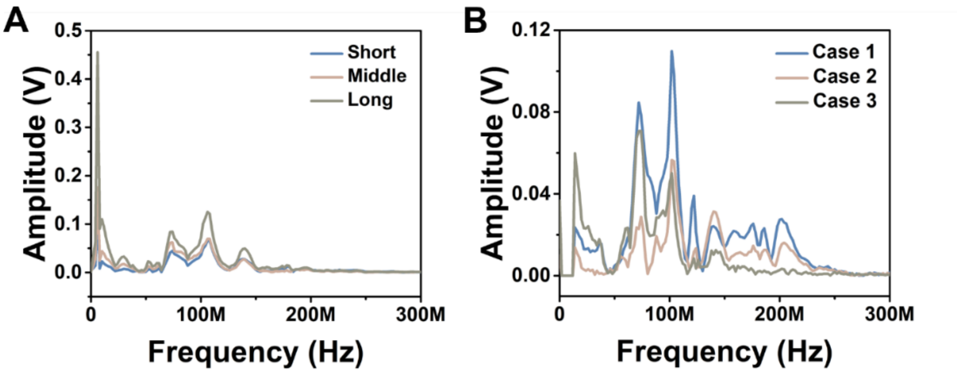

Fig. S13. The adjustable of the amplitude and spectrum of the wireless signal generated by the SWISE. (**A**) Driven by the identical mechanical motion, the larger gap distance can generate stronger signal with larger amplitude. (**B**) By adding the metal film or positioning the SWISE surrounded by external conductors, the signal spectrum changed, where the LC resonant signal was filtered by band block filter to highlight the signal.

Table S1. The major influential factors to the transmitted signal.

Influential factor	Symbol
Voltage drop on breakdown discharger	*U*
Gap distance between electrodes of breakdown discharger	*d*
Motion direction in FS-TENG	-
Wire length connecting FS-TENG and breakdown discharger	*l*
Space conductor distribution	-
Distance between the breakdown discharger and the receiver (transmission distance)	*D*
Gas type	*N*
Gas pressure	*P*
Temperature	*T*
Humidity	*H*

Note S1. The details of charged particles induced wireless EM signal.

The charged particles with an accelerated motion can generate the electric field $\boldsymbol{E}$ and magnetic field $\boldsymbol{B}$ as described below (*43*):

$$\boldsymbol{E} = \frac{q}{4\pi\varepsilon_0 c^2 r}\boldsymbol{e}_r \times \left(\boldsymbol{e}_r \times \dot{\boldsymbol{v}}\right) \tag{1}$$

$$\boldsymbol{B} = \frac{q}{4\pi\varepsilon_0 c^3 r}\dot{\boldsymbol{v}} \times \boldsymbol{e}_r = \frac{1}{c}\boldsymbol{e}_r \times \boldsymbol{E} \tag{2}$$

where the charge, vacuum permittivity, light speed, direction vector of the charged particle and distance between the observation point and charged particle can be denoted as q, ε_0, c, $\boldsymbol{e}_r$ and r, respectively. Therefore, the varying electric and magnetic fields can generate EM waves for omnidirectional propagation which were eventually received by the receiving part.

Note S2. The detailed different cases in Fig. 2H.

Case 1: The two wires were placed above the optical platform (around 6 cm) separately, with the distance around 20 cm.

Case 2: The two wires got closer with each other than case 1, with the distance around 10 cm.

Case 3: The two wires got closer with each other than case 2, with the distance around 5 cm.

Case 4: One wire was overlapped with another one.

Case 5: The two wires twisted with each other.

Case 6: The two wires were directly placed on the optical platform.

Note S3. The detailed model of the electrical model of the SWISE.

The SWISE can be simply concluded to an electrical model, as shown in Fig. 2J, where the V_i presented the output voltage of TENG, while C_i presented the capacitance of TENG. R, C and L presented the transmit system's resistor, capacitor and inductor,

respectively. The transmit system and spatial conductors' distribution nearby existed resistor, capacitor, inductor, parasitic capacitor and parasitic inductor, which can be considered as a second-order system. Similarly, the receiver system can also be considered as a second-order system with a load of oscilloscope.

Then, the breakdown signal has the relationship with

$$A_{BD} = f(\frac{di}{dt}, G(s)) = f_A(\tau, P, U, d, G(s)) \tag{3}$$

$$f_{BD} = f_f(t_r, G(s)) = f_f(\tau, P, U, d, G(s)) \tag{4}$$

where i is the current passing through the gap, t_r is the pulse current rise time, τ is the constant which is relevant to the medium characteristic, P is the gas pressure, U is the applied voltage, d is the gap distance, $G(s)$ is the transmit system characteristic, A_{BD} is the amplitude of the discharge-induced signal, and f_{BD} is the frequency of the discharge-induced signal (*44-46*).

Movie S1. The detection of the sub-finger-nail size SWISE's signal.

Movie S2. The long-distance detection of SWISE's signal.

Movie S3. The SWISE based self-powered wireless motion sensing electronic skin system.

Movie S4. The SWISE based self-powered wireless soft keyboard system.

Movie S5. The SWISE based smart wristband system.

REFERENCES AND NOTES

1. R. Haight, W. Haensch, D. Friedman, Solar-powering the Internet of Things. *Science* **353**, 124–125 (2016).
2. A. D. Maynard, Navigating the fourth industrial revolution. *Nat. Nanotechnol.* **10**, 1005–1006 (2015).
3. A. Al-Fuqaha, M. Guizani, M. Mohammadi, M. Aledhari, M. Ayyash, Internet of things: A survey on enabling technologies, protocols, and applications. *IEEE Commun. Surv. Tutor.* **17**, 2347–2376 (2015).
4. S. Li, L. D. Xu, S. Zhao, 5G Internet of Things: A survey. *J. Ind. Inf. Integration* **10**, 1–9 (2018).
5. C. Bartolozzi, L. Natale, F. Nori, G. Metta, Robots with a sense of touch. *Nat. Mater.* **15**, 921–925 (2016).
6. S. Y. Kim, Y. Choo, R. A. Bilodeau, M. C. Yuen, G. Kaufman, D. S. Shah, C. O. Osuji, R. Kramer-Bottiglio, Sustainable manufacturing of sensors onto soft systems using self-coagulating conductive Pickering emulsions. *Sci. Robot.* **5**, eaay3604 (2020).
7. T. Jin, Z. Sun, L. Li, Q. Zhang, M. Zhu, Z. Zhang, G. Yuan, T. Chen, Y. Tian, X. Hou, C. Lee, Triboelectric nanogenerator sensors for soft robotics aiming at digital twin applications. *Nat. Commun.* **11**, 5381 (2020).
8. H. U. Chung, B. H. Kim, J. Y. Lee, J. Lee, Z. Xie, E. M. Ibler, K. Lee, A. Banks, J. Y. Jeong, J. Kim, C. Ogle, D. Grande, Y. Yu, H. Jang, P. Assem, D. Ryu, J. W. Kwak, M. Namkoong, J. B. Park, Y. Lee, D. H. Kim, A. Ryu, J. Jeong, K. You, B. Ji, Z. Liu, Q. Huo, X. Feng, Y. Deng, Y. Xu, K.-I. Jang, J. Kim, Y. Zhang, R. Ghaffari, C. M. Rand, M. Schau, A. Hamvas, D. E. Weese-Mayer, Y. Huang, S. M. Lee, C. H. Lee, N. R. Shanbhag, A. S. Paller, S. Xu, J. A. Rogers, Binodal, wireless epidermal electronic systems with in-sensor analytics for neonatal intensive care. *Science* **363**, eaau0780 (2019).
9. A. J. Bandodkar, P. Gutruf, J. Choi, K. Lee, Y. Sekine, J. T. Reeder, W. J. Jeang, A. J. Aranyosi, S. P. Lee, J. B. Model, R. Ghaffari, C.-J. Su, J. P. Leshock, T. Ray, A. Verrillo, K. Thomas, V. Krishnamurthi, S. Han, J. Kim, S. Krishnan, T. Hang, J. A. Rogers, Battery-free, skin-interfaced microfluidic/electronic systems for simultaneous electrochemical, colorimetric, and volumetric analysis of sweat. *Sci. Adv.* **5**, eaav3294 (2019).
10. S. Han, J. Kim, S. M. Won, Y. Ma, D. Kang, Z. Xie, K.-T. Lee, H. U. Chung, A. Banks, S. Min, S. Y. Heo, C. R. Davies, J. W. Lee, C.-H. Lee, B. H. Kim, K. Li, Y. Zhou, C. Wei, X. Feng, Y. Huang, J. A. Rogers, Battery-free, wireless sensors for full-body pressure and temperature mapping. *Sci. Transl. Med.* **10**, eaan4950 (2018).

11. Q. Zheng, H. Zhang, B. Shi, X. Xue, Z. Liu, Y. Jin, Y. Ma, Y. Zou, X. Wang, Z. An, W. Tang, W. Zhang, F. Yang, Y. Liu, X. Lang, Z. Xu, Z. Li, Z. L. Wang, *In Vivo* self-powered wireless cardiac monitoring *via* implantable triboelectric nanogenerator. *ACS Nano* **10**, 6510–6518 (2016).
12. J. Byun, Y. Lee, J. Yoon, B. Lee, E. Oh, S. Chung, T. Lee, K.-J. Cho, J. Kim, Y. Hong, Electronic skins for soft, compact, reversible assembly of wirelessly activated fully soft robots. *Sci. Robot.* **3**, eaas9020 (2018).
13. A. D. Mickle, S. M. Won, K. N. Noh, J. Yoon, K. W. Meacham, Y. Xue, L. A. McIlvried, B. A. Copits, V. K. Samineni, K. E. Crawford, D. H. Kim, P. Srivastava, B. H. Kim, S. Min, Y. Shiuan, Y. Yun, M. A. Payne, J. Zhang, H. Jang, Y. Li, H. H. Lai, Y. Huang, S.-I. Park, R. W. Gereau, J. A. Rogers, A wireless closed-loop system for optogenetic peripheral neuromodulation. *Nature* **565**, 361–365 (2019).
14. C. Zhang, J. Chen, W. Xuan, S. Huang, B. You, W. Li, L. Sun, H. Jin, X. Wang, S. Dong, J. Luo, A. J. Flewitt, Z. L. Wang, Conjunction of triboelectric nanogenerator with induction coils as wireless power sources and self-powered wireless sensors. *Nat. Commun.* **11**, 58 (2020).
15. S. Xu, Y. Zhang, L. Jia, K. E. Mathewson, K.-I. Jang, J. Kim, H. Fu, X. Huang, P. Chava, R. Wang, S. Bhole, L. Wang, Y. J. Na, Y. Guan, M. Flavin, Z. Han, Y. Huang, J. A. Rogers, Soft microfluidic assemblies of sensors, circuits, and radios for the skin. *Science* **344**, 70–74 (2014).
16. Z. Huang, Y. Hao, Y. Li, H. Hu, C. Wang, A. Nomoto, T. Pan, Y. Gu, Y. Chen, T. Zhang, W. Li, Y. Lei, N. Kim, C. Wang, L. Zhang, J. W. Ward, A. Maralani, X. Li, M. F. Durstock, A. Pisano, Y. Lin, S. Xu, Three-dimensional integrated stretchable electronics. *Nat. Electron.* **1**, 473–480 (2018).
17. W. Gao, S. Emaminejad, H. Y. Y. Nyein, S. Challa, K. Chen, A. Peck, H. M. Fahad, H. Ota, H. Shiraki, D. Kiriya, D.-H. Lien, G. A. Brooks, R. W. Davis, A. Javey, Fully integrated wearable sensor arrays for multiplexed *in situ* perspiration analysis. *Nature* **529**, 509–514 (2016).
18. X. Hu, L. Xu, X. Lin, M. Pecht, Battery lifetime prognostics. *Joule* **4**, 310–346 (2020).
19. R. E. Ciez, J. F. Whitacre, Examining different recycling processes for lithium-ion batteries. *Nat. Sustain.* **2**, 148–156 (2019).
20. S. Niu, N. Matsuhisa, L. Beker, J. Li, S. Wang, J. Wang, Y. Jiang, X. Yan, Y. Yun, W. Burnett, A. S. Y. Poon, J. B. H. Tok, X. Chen, Z. Bao, A wireless body area sensor network based on stretchable passive tags. *Nat. Electron.* **2**, 361–368 (2019).
21. Y. Yang, Y. Song, X. Bo, J. Min, O. S. Pak, L. Zhu, M. Wang, J. Tu, A. Kogan, H. Zhang, T. K. Hsiai, Z. Li, W. Gao, A laser-engraved wearable sensor for sensitive detection of uric acid and tyrosine in sweat. *Nat. Biotechnol.* **38**, 217–224 (2020).

22. F.-R. Fan, Z.-Q. Tian, Z. Lin Wang, Flexible triboelectric generator. *Nano Energy* **1**, 328–334 (2012).
23. Z. L. Wang, Triboelectric nanogenerator (TENG)—Sparking an energy and sensor revolution. *Adv. Energy Mater.* **10**, 2000137 (2020).
24. Z. L. Wang, On the first principle theory of nanogenerators from Maxwell's equations. *Nano Energy* **68**, 104272 (2020).
25. J. Shao, M. Willatzen, Z. L. Wang, Theoretical modeling of triboelectric nanogenerators (TENGs). *J. Appl. Phys.* **128**, 111101 (2020).
26. Z. L. Wang, On Maxwell's displacement current for energy and sensors: The origin of nanogenerators. *Mater. Today* **20**, 74–82 (2017).
27. K. Dong, J. Deng, Y. Zi, Y.-C. Wang, C. Xu, H. Zou, W. Ding, Y. Dai, B. Gu, B. Sun, Z. L. Wang, 3D orthogonal woven triboelectric nanogenerator for effective biomechanical energy harvesting and as self-powered active motion sensors. *Adv. Mater.* **29**, 1702648 (2017).
28. Q. Jing, Y. Xie, G. Zhu, R. P. S. Han, Z. L. Wang, Self-powered thin-film motion vector sensor. *Nat. Commun.* **6**, 8031 (2015).
29. R. Hinchet, H.-J. Yoon, H. Ryu, M.-K. Kim, E.-K. Choi, D.-S. Kim, S.-W. Kim, Transcutaneous ultrasound energy harvesting using capacitive triboelectric technology. *Science* **365**, 491–494 (2019).
30. Y. Qi, M. C. McAlpine, Nanotechnology-enabled flexible and biocompatible energy harvesting. *Energ. Environ. Sci.* **3**, 1275–1285 (2010).
31. X. Cao, M. Zhang, J. Huang, T. Jiang, J. Zou, N. Wang, Z. L. Wang, Inductor-free wireless energy delivery via Maxwell's displacement current from an electrodeless triboelectric nanogenerator. *Adv. Mater.* **30**, 1704077 (2018).
32. S. S. K. Mallineni, Y. Dong, H. Behlow, A. M. Rao, R. Podila, A wireless triboelectric nanogenerator. *Adv. Energy Mater.* **8**, 1702736 (2018).
33. J. Kim, H. Cho, M. Han, Y. Jung, S. S. Kwak, H.-J. Yoon, B. Park, H. Kim, H. Kim, J. Park, S.-W. Kim, Ultrahigh power output from triboelectric nanogenerator based on serrated electrode via spark discharge. *Adv. Energy Mater.* **10**, 2002312 (2020).
34. P. F. Wilson, M. T. Ma, Fields radiated by electrostatic discharges. *IEEE Trans. Electromagn. Compat.* **33**, 10–18 (1991).
35. E. Sili, F. Koliatene, J. P. Cambronne, in *2011 Annual Report Conference on Electrical Insulation and Dielectric Phenomena* (2011), pp. 464–467.

36. V. Nguyen, R. Zhu, R. Yang, Environmental effects on nanogenerators. *Nano Energy* **14**, 49–61 (2015).
37. J. Fu, G. Xu, C. Li, X. Xia, D. Guan, J. Li, Z. Huang, Y. Zi, Achieving ultrahigh output energy density of triboelectric nanogenerators in high-pressure gas environment. *Adv. Sci.* **7**, 2001757 (2020).
38. K. Fujii, M. Yamada, A. Tanaka, K. Kurosawa, in *Proceedings of the Conference Record of the 1992 IEEE International Symposium on Electrical Insulation* (1992), pp. 332–335.
39. Y. LeCun, Y. Bengio, G. Hinton, Deep learning. *Nature* **521**, 436–444 (2015).
40. S. Sundaram, P. Kellnhofer, Y. Li, J.-Y. Zhu, A. Torralba, W. Matusik, Learning the signatures of the human grasp using a scalable tactile glove. *Nature* **569**, 698–702 (2019).
41. Q. Shi, Z. Zhang, T. He, Z. Sun, B. Wang, Y. Feng, X. Shan, B. Salam, C. Lee, Deep learning enabled smart mats as a scalable floor monitoring system. *Nat. Commun.* **11**, 4609 (2020).
42. K. Greff, R. K. Srivastava, J. Koutník, B. R. Steunebrink, J. Schmidhuber, LSTM: A search space odyssey. *IEEE Trans. Neural. Netw. Learn. Syst.* **28**, 2222–2232 (2017).
43. E. Landi Degl'Innocenti, in *Atomic Spectroscopy and Radiative Processes*, E. Landi Degl'Innocenti, Ed. (Springer Milan, 2014), pp. 41–83.
44. H. Okubo, N. Hayakawa, A. Matsushita, The relationship between partial discharge current pulse waveforms and physical mechanisms. *IEEE Electr. Insul. Mag.* **18**, 38–45 (2002).
45. M. Florkowski, B. Florkowska, Phase-resolved rise-time-based discrimination of partial discharges. *IET Gener. Transm. Distrib.* **3**, 115–124 (2009).
46. D. G. Kasten, X. Liu, S. A. Sebo, D. F. Grosjean, D. L. Schweickart, Partial discharge measurements in air and argon at low pressures with and without a dielectric barrier. *IEEE Trans. Dielect. Elect. Insulation* **12**, 362–373 (2005).
